# Enhancing public awareness and practice of prostate cancer screening among African men: A scoping review

**DOI:** 10.4102/safp.v65i1.5621

**Published:** 2023-01-17

**Authors:** Matthew O. Benedict, Wilhelm J. Steinberg, Frederik M. Claassen, Nathaniel Mofolo

**Affiliations:** 1 Department of Family Medicine, Faculty of Health Sciences, University of the Free State, Bloemfontein, South Africa; 2 Department of Urology, Faculty of Health Sciences, University of the Free State, Bloemfontein, South Africa; 3 School of Clinical Medicine, Faculty of Health Sciences, University of the Free State, Bloemfontein, South Africa

**Keywords:** prostate cancer, screening, knowledge, awareness, health education, African men

## Abstract

**Background:**

Prostate cancer (PCa)-related incidence is on the increase, with black South African men presenting disproportionately with aggressive disease. Recent studies show a greater net benefit of prostate-specific antigen (PSA) screening of black men compared with the general population. This scoping review provides an overview of available research on strategies that have enhanced PCa screening awareness and practice in the African setting.

**Methods:**

Relevant databases were searched to identify 2010–2021 studies. Following scoping review guidelines, data were extracted, summarised and quantitatively analysed.

**Results:**

Of the 21 articles included, 19 were from the United States. Nine were published within the last five years and 14 were pre-test/post-test. Most articles evaluated the effect of health-promoting strategies on awareness and practice of PCa screening among black men. Community-oriented strategies enhanced awareness and practice of PCa screening. Healthcare providers and community members, including PCa survivors, participated in the strategies’ planning, development and implementation. Topics that improve PCa knowledge and clear cultural misconceptions were addressed, targeting public spaces unique to these men. Prostate cancer health education methods were diverse, comprehensive, user friendly and culturally sensitive.

**Conclusion:**

More research on strategies to enhance PCa screening awareness and practice among African men is needed, as this is scarce. Strategies enhancing PCa screening awareness and practice among African men are community oriented and entail health education methods, topics, presenters and venues. These strategies can be adopted in the South African setting.

**Contribution:**

This study recommends strategies to enhance the awareness and practice of PCa screening among African men.

## Introduction

Prostate cancer (PCa) is the fourth most commonly diagnosed cancer worldwide and ranks the second most frequently diagnosed malignancy (after lung cancer) in men worldwide.^1^ Evidence shows higher mortality rates from PCa among black African populations than other races. This disproportionate mode of presentation has also been described between regions in Africa. For example, reports show lower PCa incidence and mortality rates of 10.6 and 7.0 per 100 000 men in northern Africa, compared with the average rates of 34.3 and 22.1 per 100 000 men observed in sub-Saharan Africa (SSA). Factors attributable to these variations include poor socioeconomic status, dietary differences, genetic differences and the presence of infectious diseases in SSA.^2^ Other socioeconomic and health systems factors attributable to the variance in PCa presentation include the absence of low-cost, community-based screening and health promotion programmes, late presentation of patients to healthcare facilities, fewer treatment options, lack of adequate follow-up and inherent social norms and beliefs.^3^ Reasons for the late presentation of men, often with advanced disease, include low health literacy and the cultural concept of the disease, masculinity and disease, poor help-seeking behaviour and failure to take the sick role.^4^ Despite the racial disproportion in the morbidity and mortality from PCa, black men are underrepresented in diagnostic and therapeutic clinical trials on this disease.^2^

The incidence of PCa in South Africa is on the upward trend: 29.4 per 100 000 men in 2007 and 67.9 per 100 000 men in 2012.^5^ In South Africa, PCa has the highest frequency of all new cancer cases, with 3896 deaths occurring of the 13 152 new patients (29.6%).^6^ Compared with men of other races, South African black men present with higher prostate-specific antigen (PSA) levels and higher stage and grade of disease, with the majority of the cases being incurable. Also, compared with African American men, South African black men had a higher prevalence of aggressive disease and higher PSA levels, especially those from rural communities.^7^

Delayed diagnosis has been identified as a likely cause for patients presenting with advanced disease. The mean age of black patients at presentation in two South African studies (KwaZulu-Natal and the Western Cape) was 71.6 and 68.9 years, respectively.^5^ This was higher than the mean age of 64.7 years in black patients in the United States Surveillance Epidemiology and End Results (SEER) database. Reasons identified for delayed diagnosis among the patients in a South African study^5^ were a lack of PCa screening programme and a referral population that is primarily rural, with poor socioeconomic status, high unemployment rates, and low literacy and education levels. Another contributing factor may be adherence to a strong traditional belief system, with patients preferring to visit traditional healers rather than a clinic or hospital.^5^ Many obstacles to accessing health services exist in poor rural communities, resulting in lost-to-follow-up.^5^

A study was performed in the Free State to determine the profile and risk stratification of patients with PCa treated at the Department of Oncology, Universitas Annex in Bloemfontein.^8^ The majority (72.8%) of the patients were of the black race, 43.7% had high-grade disease (i.e. Gleason score 8–10), 67.9% had PSA levels > 20 ng/mL and 62.3% had T stage ≥ T3. Nearly half the patients (48.7%) had stage IV disease, and 38.4% received palliative hormonal therapy as initial treatment. The majority of the patients (82.5%) fell into the high-risk group.

### Prostate cancer screening

Prostate cancer screening is an attempt to diagnose PCa in asymptomatic men through digital rectal examination (DRE) and the measurement of serum PSA. Despite the inconclusive benefits of using DRE and PSA as screening tests, large population-based studies showed increased survival benefits in the early treatment of PCa compared with no active therapy in men with moderately and poorly differentiated disease.^9^

The United States Preventive Task Force (USPSTF) has reported a potential benefit of decreasing deaths from PCa in men aged 55–69 years with PSA screening. However, the benefit of screening is doubtful for men above 70 years of age.^10,11,12^ The relatively higher mortality from PCa observed in Africa has been partly attributed to the limited availability of screening and early detection.^13^ Although PCa screening remains controversial – it is currently a method recognised to control PCa disease through early detection.^14^

According to the American Cancer Society, there is evidence that PSA screening can detect early-stage PCa, and it is recommended that men at high risk, based on race and family history, should begin early detection with a PSA blood test and DRE at 45 years of age. Men at higher risk (having more than one first-degree relative who had cancer at an early stage) should start early detection at 40 years of age.^15^

Overdiagnosis and overtreatment are the major challenges associated with PSA screening. A diagnosis of PCa usually calls for treatment, and all PCa treatments carry a considerable risk of side effects such as sexual and urinary dysfunction. In as much as diagnosing aggressive cancers can be lifesaving, diagnosing harmless cancers does more harm than good.^16^ A recent European study on the harm to benefit of PCa screening in black men showed that although the potential for overdiagnosis and overtreatment remains, the net benefit of PSA screening is greater for black men than the general population. Therefore, policymakers may need to consider the need for race-specific screening guidelines.^2^

In South Africa, PCa screening is equally common across all race groups. South African guidelines^17^ recommend that PCa screening be performed in all men from 45 years onwards and in the absence of identifiable risk factors, from 40 years in black African males, where there is a family history of PCa and other identifiable risk factors. In South Africa, PCa screening involves both DRE and PSA.^17^ The recommended guidelines notwithstanding, the uptake of PCa screening is low among African men, which raises a suspicion of patterns in knowledge and beliefs towards PCa screening within a subculture.^15^ The higher mortality from PCa observed among African men may be a consequence of their screening behaviour.

In as much as it might be financially challenging to screen the entire population for PCa, once a patient deemed to belong in the high-risk category attends a doctor with a complaint (the nature notwithstanding), a simple screening process can be incorporated into the consultation with little extra time or effort.^18^ As stated by Glynn:^18^

In health economic terms, a true screening programme for a particular disease across a whole population has to be evaluated as being useful, economic and with no negative effects. (p. 8)

To address the quadruple burden of disease in South Africa, the focus has been on maternal and child (health) care, HIV and pulmonary tuberculosis, trauma and non-communicable diseases.^19^ Under-representation of male-related diseases within the healthcare systems and the media has been observed. Even though South Africa has national registries for breast and cervical cancers, none exists for PCa.^20^ There seems to be a lack of prioritisation and limited secondary prevention strategies geared towards PCa disease.

### Arguments for an enhanced public awareness of prostate cancer screening

Studies have identified the lack of knowledge on PCa among African men.^21,22^ In fact, most South African indigenous languages do not have translations for the term ‘prostate gland’. The racial difference in stage at presentation and therapy shows the need for greater PCa awareness and education among patients and primary healthcare practitioners. Early detection of PCa through more widespread PSA screening may be of value among this identified vulnerable group of men.^21,23^

Among men with some degree of PCa knowledge, there is still a low turn up for screening for this disease. A Kenyan study^13^ showed that the screening rate is still low despite massive education campaigns on PCa awareness in Kenya. Apart from knowledge gaps, certain cultural and belief factors were responsible for the low turn up for PCa screening.^13^

An Asian study^24^ showed that health promotion plans and educational intervention programmes provided by healthcare practitioners would increase awareness and correct false impressions about PCa, ultimately stimulating screening among men. Education programmes should be designed to identify and correct public misrepresentations for individuals to recognise health concerns and gain more knowledge. The study further showed that educational videos were significantly effective in motivating and educating participants about their beliefs on PSA screening for early detection of PCa.^24^

Locally in South Africa, arguments exist for enhanced health awareness through community participation. The Patients’ Rights Charter of South Africa^25^ is a charter of the National Department of Health that promotes and protects patients’ rights in the healthcare sector. This charter affords patients the right to participate in healthcare decision-making, access healthcare, confidentiality and privacy, informed consent, refuse treatment and the continuity of care.^25^ The South African ‘Batho Pele Principles’^26^ serve as an acceptable policy and legislative framework regarding service delivery in the public service. These principles align with the constitutional ideals of providing service impartially, utilising resources efficiently and effectively and responding to people’s needs through participation. One of the prime aims of Batho Pele is to provide a framework geared at increasing access to healthcare information and services to the many South Africans who do not have such access.^26^

Hence, studies have shown gaps in the awareness and practice of black men regarding PCa screening; however, there is a paucity of studies on the specific strategies to close these identified gaps among this vulnerable group of men.

The aim of this scoping review was to provide an overview of the available research on strategies proven to have enhanced PCa screening awareness and practice in the African setting. The following research question was formulated: What health-promoting strategies have been shown to improve African men’s awareness and practice of PCa screening?

## Methods

### Study design

This was a scoping review; the details of the conduct are described next.

### Inclusion and exclusion criteria

The conduct of this review adhered to the Preferred Reporting Items for Systematic Reviews and Meta-Analyses – Extension for Scoping Reviews (PRISMA-ScR)^27^ and Joanna Brigg’s Institute guidance for the conduct of scoping reviews.^28^ To be included in the review, articles needed to: (1) be about humans (of African descent), (2) have been conducted in an African setting, (3) be in English, (4) be related to focus on PCa health-promoting strategies and (5) have been published during the past 11 years (up to and including 2021). No limits were placed on the type of article.

### Search strategy

To identify potentially relevant documents, the following search terms were used to search for the evidence:

- ‘primary health*’ and (Knowledge or attitude* or practice*) and (Screen* or psa or antigen* or ‘rectal* exam*’ or ‘physical* exam*’ or ‘early diagnos*’) and ‘Prostate cancer’ and (‘African men’ or ‘black men’)
- (Screen* or psa or antigen* or ‘rectal* exam*’ or ‘physical* exam*’ or ‘early diagnos*’) and ‘Prostate cancer’ and (‘African men’ or ‘black men’) and ti (african* or black*)
- ((educat* or Teach* or learn* or train* or inform*) n3 (tool* or method* or interven*)) and ‘prostate cancer’ and (Screen* or psa or antigen* or ‘rectal* exam*’ or ‘physical* exam*’ or ‘early diagnos*’) and (kap or knowledge or attitude* or practice*)
- ((educat* or Teach* or learn* or train*) and (black* or african*) and ‘prostate cancer’ and (Screen* or psa or antigen* or ‘rectal* exam*’ or ‘physical* exam*’ or ‘early diagnos*’)) not ‘african american’

Searches were conducted in the following databases from 2010 to 2021: Academic Search Ultimate, Africa-Wide Information, APA PsycArticles, APA PsycInfo, CINAHL with Full Text, Communication & Mass Media Complete, ERIC, Health Source – Consumer Edition, Health Source: Nursing/Academic Edition, Humanities Source Ultimate, MEDLINE, Sociology Source Ultimate, MasterFILE Premier. The first searches were conducted on 14 August 2020 while the last ones were conducted on 31 August 2021. The search strategy was conducted by the principal researcher with the assistance of a senior university librarian.

### Screening and selection of articles

Each researcher screened the titles and abstracts to exclude studies that fell short of the inclusion and exclusion criteria. The researchers sent their selected studies to the principal researcher, who excluded duplications and combined the studies into a single list. This list was sent to the librarian who obtained the full-text articles. Full-text articles that fell short of the inclusion and exclusion criteria were excluded.

### Data synthesis

The researchers extracted data using a standardised template (data charting form) developed by two of the researchers. The template included the first author, year of publication, country of origin, publication type, study design, aim of the study, strategies engaged and key findings. Each researcher submitted the completed templates to the principal researcher, who integrated them into one document. As this was a scoping review, no critical appraisal of the quality of the included articles was performed.

### Ethical considerations

The study was approved by the Health Sciences Research Ethics Committee (HSREC) of the University of the Free State (ref. no. UFS-HSD2020/1481/2411). Permission to conduct the study was granted by the Head of the Free State Department of Health.

## Results

**FIGURE 1:**
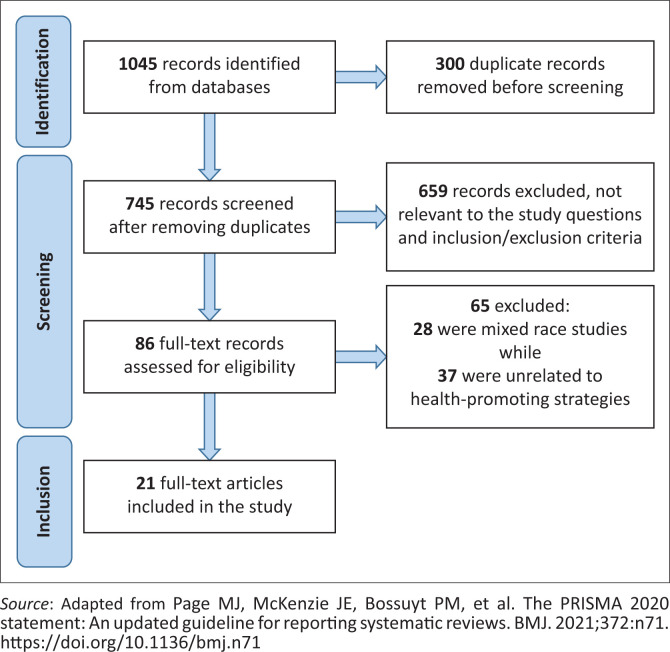
Flow diagram of article selection.

The search strategies yielded 1045 publications. Following deduplication (300 duplicates removed), 745 publications were left. After screening by title and abstract, 659 publications were neither specific in terms of population nor context. The full texts of the remaining 86 articles were assessed for eligibility. Sixty-five full-text articles were further found irrelevant; 28 were mixed race studies, while 37 had concepts unrelated to health-promoting strategies, leaving 21 articles in the review. Figure 1 is a flow diagram of article selection.

### Characteristics of the included articles

A total of 21 published articles were included in the review, the majority (90.5%) of which were from the United States (Table 1). About a third were published within the last five years. Two-thirds (*n* = 14) of the articles were pre-test/post-test in nature. All but one (PhD thesis) of the included articles were original research. Most articles aimed to evaluate the effect of certain health-promoting strategies on the awareness and practice of PCa screening among black males.

**TABLE 1:** Studies included in the scoping review.

	Author	Year	Country	Publication type and study design	Aim of the study and strategies engaged	Key findings of the study
1	Howard et al.^29^	2018	US	Original research Pre-test/post-test survey of black men aged ≥ 40 years	**Aim:** To provide black men with a culturally targeted faith-based prostate cancer (PCa) education programme and evaluate its efficacy **Strategies:** Education programmes directed at church members in a rural community. Black survivors of PCa motivated the community through their live and video-clip testimonies. Live teachings on PCa statistics, prevention, screening, early detection, treatment and quality of life given by physicians.	The PCa knowledge improved Increased intention to have shared decision-making (SDM) with a physician within 12 months The actual practice of SDM with a physician improved within 3 months
2	Hunter et al.^30^	2015	US	Original research Grounded theory qualitative research design	**Aim:** To describe African Americans’ perceptions and beliefs regarding PCa risk and (prostate-specific antigen) PSA testing **Strategies:** Participants were recruited from rural community health centres (CHC) and churches and comprised men and women who were lay health advisors, PCa survivors (and their loved ones) and others interested in the health of their community. PowerPoint presentation on steps to informed decision-making (IDM) for PCa screening. Thereafter, participants were engaged in conversation with one another about issues related to prostate health.	Improved knowledge of the benefits of PCa screening Intention to have PCa screening increased Involvement of trained female lay health advisors perceived to be effective in helping men obtain prostate care Community outreach, using culturally sensitive educational materials often effective
3	Lepore et al.^31^	2012	US	Original research Randomised controlled trial design with pre-test/post-test measures	**Aim:** To evaluate the efficacy of a decision support intervention focused on PCa screening among certain immigrant black men **Strategy:** Decision support intervention using educational pamphlets and tailored telephone education on PCa testing.	Better knowledge of PCa and PCa screening Decreased PCa screening decision conflict Increased first-time SDM with physicians No effect on intentions to get PCa test
4	Odedina et al.^32^	2014	US	Original research Pre-test/post-test survey of black men	**Aim:** To evaluate the effect of a culturally relevant video intervention on the prostate health behaviour of black men **Strategy:** A barbershop served as the venue for this intervention. Video starring PCa survivors, PCa advocates, a radio personality and barbers. Motivational remarks were provided by a PCa scientist, a urologist, a PCa advocate, a former legislator and a minister.	Increased PCa knowledge Decreased decisional uncertainty Intention to participate in PCa screening increased
5	Patel et al.^33^	2013	US	Original research Pre- and post-interventional structured interview of focus groups	**Aim:** To evaluate the effect of an educational intervention on IDM on PCa screening, screening rates and PCa knowledge among low-income African American men **Strategy:** Intervention was delivered by trained lay community educators using a PCa educational brochure developed with the community. Focus group discussion (FGD) involving males ≥ 40 years who had not had PSA screening in the past year, those who had been screened in the past year and their family members and significant others.	Average knowledge scores increased Higher PSA screening rate
6	Troy et al.^34^	2020	US	Original research Pre-test/post-test survey	**Aim:** To assess the effects of certain educational programmes on PCa knowledge and decision-making **Strategy:** African American men (and their partners/relatives) were recruited for a PCa education programme using flyers, radio and newspaper advertisements, professional listservs and word-of-mouth. Culturally appropriate and community-tailored education programmes were engaged. Presenters included a PCa survivor, registered dietitians, physical activity experts and a cancer communication researcher. Workgroup members included university researchers, community members, PCa survivors and clinicians. A PCa survivor and healthcare professionals administered this education programme.	The PCa education and awareness improved
7	Frencher et al.^35^	2016	US	Original research Non-randomised pre-test/post-test comparison study	**Aim:** To investigate the effect of decision support instruments (DSIs) in the form of a video show on African American men’s PCa screening decision **Strategy:** Barbershop-based health education in an African American community. The DSIs were culturally tailored, and their effects were compared with those of culturally non-specific DSIs.	The PCa knowledge increased following exposure to both DSIs in equivalent proportions The culturally-tailored DSI demonstrated an increase in screening intention Increased certainty in the decision-making process about PCa screening Increased rate of PSA testing
8	Livingston et al.^36^	2018	US	Original research Retrospective pre-post survey design	**Aim:** To assess the effectiveness of educational workshops on black American men’s knowledge and health behaviour related to PCa **Strategy:** The FGD and interviews involving cancer survivors, an oncologist, cancer researchers and community health workers to identify barriers to black men’s participation in PCa educational workshops. The PCa educational workshops were presented.	Increased belief in the importance of PCa screenings for black American men Positive changes in health behaviour and intentions; greater ease in arranging PCa screening, and greater ease in following through with appointments Improved SDM knowledge
9	Capanna et al.^37^	2015	Jamaica	Original research Pre-test/post-test design	**Aim:** To evaluate the impact of a theory-based health education intervention on awareness of PCa and intention to screen among African men **Strategy:** Health education intervention was administered by a study staff member using a PowerPoint presentation. Topics presented included basic anatomy and physiology of the prostate gland and easy descriptions of its location on a man’s body, how PCa spreads, risk factors, signs and symptoms and all possible PCa screening methods. Follow-ups through phone 6 months after the intervention	Increased PCa knowledge Increased PCa screening rate within 6 months post-intervention
10	Carter et al.^38^	2010	US	Original research Quasi-experimental three-phase intervention programme	**Aim:** To determine the effectiveness of an educational intervention programme on PCa screening behaviour **Strategies:** Three-phased community-based intervention programmes: focus groups, education and follow-up. Each phase provided information on PCa, PCa screening and the importance of early detection. The focus group included female participants.	Improved knowledge of PCa screening and the importance of early detection Increased PCa screening rates Women play an important role in supporting male participation in PCa screening
11	Holt et al.^39^	2017	US	Original research A cluster randomised design	**Aim:** To determine the effect of female health partner involvement on PCa screening IDM **Strategies:** Quarterly workshops on IDM were conducted in the form of didactic and interactive discussions, stories and testimonies. Baseline IDM outcomes were measured after the first workshop and compared with outcomes of subsequent workshops. Comparing an all-men group with a mixed-gender group to determine the effect of female health partner’s involvement on IDM outcomes.	Multiple interventions (workshops) resulted in better IDM outcomes Involvement of female health partners showed no change in IDM outcome Lower decisional conflict was evident by an improved decision-making stage Preference in participating in decision-making as against reliance on doctor at baseline
12	Jones-Dendy et al.^40^	2017	US	Thesis (PhD) A mixed method study (randomised controlled trial and focus group discussion)	**Aim:** To determine the effect of combining a standard PCa education video with a testimonial presentation from a community PCa lay survivor on PCa knowledge and self-efficacy for IDM **Strategies:** Combination of a standard PCa education video with a testimonial presentation from a community PCa lay survivor. Participants were recruited from local churches and barbershops.	Both PCa knowledge and self-efficacy for IDM improved with the combined intervention
13	Luque et al.^41^	2015	US	Original research Structured interview Pre-test/post-test survey	**Aim:** To explore the feasibility and receptiveness of African American barbers to become barber health advisors and partner with researchers to test the efficacy of a PCa education intervention programme	Rural African American barbershops are appropriate and feasible settings for PCa health promotion Barbers can complete a training curriculum on PCa and gain the knowledge to become barber health advisors
14	Luque et al.^42^	2011	US	Original research Post-intervention survey (post-test only) to determine the impact of an educational intervention	**Aim:** To assess the effect of barbershop-based PCa education **Strategies:** Barbershops were used as platforms for PCa health education, where the barbers were the instructors. The barbers were trained using a combination of the following methods: didactic instruction, interactive group exercises and team building, congruent with Empowerment Education learning approaches. Mode of instruction included brochure, video and flipcharts.	Increased self-reported knowledge of PCa among barbershop clients Increased likelihood of clients discussing PCa screening with a healthcare provider
15	McCree-Hale et al.^43^	2012	Jamaica	Original research Cross-sectional pre-test/post-test survey	**Aim:** To evaluate the impact of a theory-based health education intervention among men in Western Jamaica **Strategies:** Theory-based patient education programme. PowerPoint presentation. The use of basic illustrations of PCa screenings and culturally relevant media, including pictures.	Increased PCa screening knowledge Increased intention to screen
16	Okoro et al.^44^	2018	US	Original research Mixed-method design	**Aim:** To assess PCa knowledge and explore perceptions on the role of women in PCa prevention **Strategies:** The FGD, including female participants.	The FGD suggested that: Involvement of community members in health education; awareness campaign is crucial Print, electronic, social and billboards were suggested media for PCa health promotion ‘Women can make a significant contribution towards reducing PCa mortality among men by being educators and an information resource, ensuring healthier living, facilitating regular primary care and generally being supportive’
17	Owens et al.^45^	2016	US	Original research Qualitative study (FGD)	**Aim:** To assess the appropriateness of a computer-based IDM intervention for PCa screening among African American men	PCa screenings were either received on the recommendation of a doctor or as a job requirement The use of technology is appropriate in the dissemination of PCa information and in preparing men for IDM
18	Ross et al.^46^	2010	US	Original research Pre-test/post-test survey	**Aim:** To evaluate the applicability of an evidence-based video intervention to promote IDM for PCa screening among African American men with different levels of health literacy **Strategy:** The use of a video to present PCa health information.	Video intervention is suitable for use among African American men with different levels of health literacy
19	Sandiford et al.^47^	2016	US	Original research Pre-test/post-test survey	**Aim:** To determine the effect of an educational intervention on PCa knowledge and SDM **Strategy:** A PowerPoint presentation of PCa health education. Involvement of church lay leaders and pastors in the planning, development and implementation of the intervention. Topics presented included incidence and mortality rates, risk factors, screening guidelines, benefits versus risks of PSA testing, survival rates, treatment options and prevention strategies. Video featuring two African American PCa survivors.	Increased PCa knowledge, awareness of personal risk and intention to participate in SDM
20	Sultan et al.^48^	2014	US	Original research Pre-test/post-test survey	**Aim:** To assess the effect of a mobile tablet-mediated intervention on PCa knowledge and screening decision-making **Strategy:** Delivery of PCa health information using internet-enabled mobile tablet technology.	Improved PCa knowledge and decisional self-efficacy Reduced decisional conflict
21	Wray et al.^49^	2011	US	Original research Pre-test/post-test survey	**Aim:** To assess the effect of an educational outreach strategy on PCa knowledge, screening barriers and decisional self-efficacy **Strategy:** Content experts, health educators and community members contributed to developing the outreach curriculum. PowerPoint presentation by public health professionals and PCa survivors. Cultural appropriateness and ease of understanding were ensured.	Increased PCa knowledge and decisional self-efficacy Decrease in barriers to screening No changes in perceived subjective norms and perceived benefits of screening were observed

CHC, community health centres; DSI, decision support instrument; IDM, informed decision-making; FGD, focus group discussion; PCa, prostate cancer; PSA, prostate-specific antigen; SDM, shared decision-making; US, United States.

### Strategies engaged and assessed in the studies

As shown in Table 2, the strategies engaged in the studies reviewed were classified under the following themes: (1) methods of health education, (2) health education topics, (3) presenters of the health education and (4) venue of the health education.

**TABLE 2:** Strategies engaged to enhance prostate cancer awareness and practice.

‘HOW’ health education	‘WHAT’ health education	‘WHO’ health education	‘WHERE’ health education
Live and recorded video clips	The PCa statistics, for example, incidence, survival and mortality rates	Black PCa survivors and their loved ones	Churches
Live teachings	Prevention, screening and early detection	Physicians/clinicians	Community health centres
Live and recorded motivational talks	Treatment options	Male and female lay advisors	Barbershops
PowerPoint presentation	Quality of life	Trained lay community educators	Civic and social organisations
Group discussion on issues relating to prostate health	Steps to informed decision-making for PCa screening	Any other community member interested in her or his community’s health	Rural community
Culturally sensitive education material	Shared decision-making	Trained barbers	-
Educational pamphlets	Anatomy and physiology of the prostate	Radio personalities	-
Tailored telephone education	Prostate location in the body	Former legislators/ministers	-
A PCa educational brochure	How PCa spreads	Physical activity experts	-
Flyers, flipcharts	Risk factors for PCa	University researchers	-
Culturally tailored decisional support instruments	Signs and symptoms of PCa	Registered dieticians	-
Repeated educational workshops	Screening methods	Community health workers	-
Didactic and interactive focus group discussion	Screening guidelines	Cancer researchers	-
Use of electronic materials	Benefits versus risks of prostate specific antigen testing	Church lay leaders/pastors	-
Computer-based informed decision-making	-	Public health professionals	-

PCa, prostate cancer.

Prostate cancer health education programmes were targeted primarily at rural community members. African American men (and their partners/relatives) were recruited for a PCa education programme using flyers, radio and newspaper advertisements, professional listservs and word of mouth.^33,34^

Both healthcare professionals and lay members of the communities were involved in the planning, development and implementation of the intervention.^29^ These health education interventions were mostly culturally targeted and faith based.^34,35,43,49^ The venues used, as found by this review, were churches,^29,30,38,40,47^ barbershops,^32,35,40,41,42^ community health centres (CHCs),^30^ rural community^29^ and other civic and social organisations.^37,38^

Motivational talks were delivered through live and video-clip testimonies of black survivors of PCa.^29,32,40,42,46,47^

Barbers were trained using a combination of didactic instruction, interactive group exercises and team building. Subsequently, they served as PCa health instructors to their male clients.^41,42^

Other community members involved in delivering motivational talks and health education comprised men and women who were church lay leaders and pastors,^47^ lay health advisors,^30^ trained lay community educators,^33^ PCa advocates, radio personality, barbers, a former legislator, a minister,^32^ registered dietitian, physical activity experts and others interested in the health of their community.^34^

The various methods of instruction engaged by these lay community members included educational pamphlets,^31^ flipcharts,^42^ culturally relevant media (e.g. pictures),^43^ educational brochures,^42^ tailored telephone education^31^ and internet-enabled mobile tablet technology.^48^

Live teachings were given to men in the community by physicians,^29^ PCa scientists and urologists,^32^ university researchers and other relevant clinicians.^34^ These teachings covered PCa statistics such as incidence, mortality and survival rates.^29,47^ Other aspects of PCa covered were basic anatomy and physiology of the prostate gland, easy description of its location in a man’s body, description of PCa and how it spreads, risk factors, signs and symptoms,^37^ prevention strategies,^47^ early detection^29,38^ and a description of all possible PCa screening methods,^37^ benefits versus risks of PSA testing,^47^ informed decision-making (IDM) for PCa screening,^30^ treatment options^47^ and quality of life.^29^

Health education methods engaged by these healthcare providers included PowerPoint presentations,^30,37,43,47,49^ focus groups (including female participants)^33,36,38,40,44^ and educational workshops^36,39^ conducted in the form of didactic and interactive discussions, stories and testimonies.

Focus groups and interviews were conducted among community stakeholders such as cancer survivors, oncologists, cancer researchers and community health workers to identify barriers to black men participating in PCa educational workshops and other issues related to prostate health.^13^

### Outcome of the strategies

The effect of the various interventions is listed in Table 3.

**TABLE 3:** Outcome of the strategies.

No.	Outcome of the strategies
1	Improved PCa knowledge and awareness
2	Increased knowledge of SDM and informed decision-making
3	Increased intention to have SDM
4	The practice of SDM with physician improved
5	Improved knowledge on benefits of PCa screening
6	Intention to have PCa screening increased
7	Decreased PCa testing decision conflict/decisional uncertainty
8	Positive changes in behavioural efficacy, for example, greater ease in arranging schedules to make time for PCa screening
9	Increased knowledge about personal risk for PCa
10	Decreased barriers to PCa screening

PCa, prostate cancer; SDM, shared decision-making.

Overall, PCa knowledge improved in 13 (61.9%) studies.^29,30,31,32,33,34,35,37,38,39,40,42,47,48,49^ There was an increase in the knowledge of informed and shared decision-making (SDM); the intention to have SDM with healthcare providers,^29,36,42,47^ while its actual practice also increased.^29,31^ Multiple interventions (workshops) resulted in better IDM outcomes.^39^ Both PCa knowledge and self-efficacy for IDM improved with the combined intervention compared with the standard education video alone.^40^ Community outreach using culturally sensitive educational materials was shown to be effective.^30^ In one of the studies, the mean knowledge score increased in 12 of the 19 knowledge items.^34^

There was an improvement in knowledge of the personal risk for PCa and the benefits of PCa screening, with a consequent decrease in PCa testing decision conflict/decisional uncertainty.^30,31,47^ There were positive changes in behavioural efficacy as well as a decrease in barriers to screening.^36,49^

Ultimately, both the intention to have a PCa screening and the actual PSA screening rate increased in seven studies.^32,33,35,37,38,43,47^ For instance, the percentage of men willing to get a PSA test within the next 12 months increased from 57% to 73% in one of the studies.^35^ However, there were contrary findings in two studies: one showed no effect on the intentions to get a PCa test,^31^ while the other showed no changes in perceived subjective norms and perceived benefits of PCa screening.^49^

Barbers were able to complete a training curriculum on PCa and gain sufficient knowledge to become barber health advisors.^41^ There was an increase in self-reported knowledge of PCa among barbershop clients; rural barbershops were both appropriate and feasible settings for PCa health promotion.^42^

Regarding the role of women in prostate health, the involvement of trained female lay health advisors was perceived to be effective in helping men obtain prostate care. Also, women seem to play an important role in supporting male participation in PCa screening.^30,38,44^ However, one study showed no change in IDM outcome, despite the involvement of female health partners.^39^

Conclusions from the focus group discussions include the following:

- Involvement of community members in health education and awareness campaign is vital^33,40,44^
- Print, electronic, social and billboards are important media for PCa health promotion^44^
- Women can make a significant contribution towards reducing PCa mortality among men by being educators and an information resource, ensuring healthier living, facilitating regular primary care and generally being supportive^44^
- Technologies should be considered for use in the widespread dissemination of PCa information and preparing men for making informed PCa screening decisions with their doctor^45^
- Video intervention is suitable for use with men with different health literacy characteristics.^32,46^

## Discussion

More than 90% of studies included in this review were conducted among African Americans in the United States, despite efforts in the search strategies to identify other non-American studies among African men, thus indicating a paucity of research on this subject among black men of African nationalities.

One of the key findings from this review was that both healthcare professionals and lay members of the communities were involved in the planning, development and implementation of the intervention. This aligns with primary healthcare initiatives, which allow for the full participation of community members in implementation and decision-making.^50^ Social participation, an essential feature in the WHO’s Framework on Integrated, People-centred Health Services, strengthens and promotes health governance.^50^ Furthermore, the community-oriented primary care (COPC) cycle involves the determination and implementation of adaptive action plans to address individual, family and community health issues; this is followed by monitoring, evaluation and review of engaged activities and plans.^51^ Finally, community involvement in the planning, development and implementation of healthcare intervention will ensure the incorporation of its cultural sensitivity, thereby enhancing its comprehension and acceptability and minimising disparity in healthcare.^52,53^

Similar to the findings of this review, churches, CHC and barbershops are also appropriate venues for PCa education and health promotion among men in South Africa. In addition, venues applicable to the South African context in the target of men for PCa health promotion include taxi ranks, physical activity/wellness centres (e.g. gym) and social gatherings, for example, barbecues. Subsequently, the owners and those in charge of these various establishments can be trained to become health education providers. In South Africa, community health workers are a vital cadre of healthcare providers conveying healthcare education and services directly to households in the community. Also, because of accessibility and affordability, up to 80% of South Africans consult traditional and spiritual healers for their primary healthcare needs.^54^ Proper training of these healthcare providers on this subject will enhance the dissemination of health education and a rational approach to PCa screening in the community. As found by this review, other community-based and academic institutional health promoters also apply to the South African context.

Globally, social media platforms such as Facebook, YouTube, Instagram, Pinterest, Twitter and WhatsApp have become effective mediums of communication with the populace.^55^ The use of social media platforms has been shown to positively influence awareness of public health behavioural changes, as seen in the current coronavirus disease 2019 (COVID-19) pandemic.^56^ Its use in disseminating health education is not without limitations and disadvantages; misleading information can also be easily circulated. Other limitations to the use of social media include inequality, the digital divide, language barriers, gender gaps, privacy concerns, age, cultural beliefs and disability.^55^ In South Africa, MomConnect, a mobile health programme initiated by the National Department of Health targeted at improving antenatal and maternal health, is one of the largest mobile health initiatives in the world. It is effective in the widespread dissemination of relevant health education to over half a million users. Such information includes nutrition, diet, drug use, warning signs for medical issues, foetal development, postnatal care and social support.^57^ Its helpdesk feature allows registered users to ask maternal and child health-related questions and provide feedback on health services received at public health clinics.^58^ Similar mobile health programmes can be developed to promote PCa-related health education among relevant men.

With the controversies surrounding PCa screening, SDM has become a crucial subject to be considered during community health education and awareness. The competence of healthcare providers in the practice of SDM is equally important. As shown in Table 2 and Table 3, SDM was one of the PCa health education topics presented to men in the community. Consequently, the knowledge, intention and practice of SDM improved. There was also a decrease in PCa testing decision conflict.

Effective health education may pose greater challenges in regions with low knowledge and literacy levels; for instance, most indigenous languages in South Africa do not have a translation for the prostate gland. Health education topics such as anatomy (location of the prostate gland) and physiology of the prostate are therefore critical in this setting. Also, interpretation and translation of health education into the common indigenous languages are essential.

### Study’s strengths and limitations

As far as we know, this seems to be one of the few studies focused on strategies that have enhanced PCa screening awareness and practice, particularly in the African setting. Strategies identified by this study may also be adapted to promote awareness and practices related to other common cancers in the study setting.

However, the following limitations relate to the study and should be borne in mind: Only studies conducted among men of African origin were included; therefore, relevant strategies promoting awareness and practice of PCa screening among other races might have been missed. Also, only articles written in English were included; relevant articles from different languages might also have been missed. Most of the studies in this review were of a pre-test and post-test study design; the positive impact revealed cannot be guaranteed in the long term as the post-tests were carried out within 6–12 months of the intervention. There is, therefore, the need for the continuity of these health-promoting strategies. Finally, attempts to find and include relevant grey literature were to no avail. Hence, only peer-reviewed articles from academic journals were included in this report.

## Recommendations

- More research on this subject should be encouraged, as they are scarce in the African settings.
- Strategies shown to have enhanced the awareness of PCa screening are community oriented, thus necessitating the involvement of healthcare providers, community members and PCa survivors in the planning, development and implementation of applicable strategies.
- Topics that improve knowledge and clear cultural misconceptions regarding PCa should be addressed, bearing in mind the public spaces unique to these men.
- Prostate cancer health education methods should be diverse, comprehensive, user friendly and culturally sensitive, with the availability of translations into the local languages.
- Similar strategies can be considered in the South African setting, bearing in mind the needful socio-cultural adaptations.
- The government and relevant parastatals should mandate and support family physicians, who are the most senior clinicians in primary healthcare settings, to champion these health promotion strategies.

## Conclusion

This scoping review sought available research on strategies that have enhanced PCa screening awareness and practice in the African setting. More research on this subject is needed as they are scarce. Strategies enhancing PCa screening awareness and practice among African men are community-oriented and entail considering relevant health education methods, topics, presenters and venues. These strategies can be adopted in the South African setting.
